# *N*-(4-Hydr­oxy-3-methoxy­benz­yl)benzamide

**DOI:** 10.1107/S1600536809027500

**Published:** 2009-07-18

**Authors:** Liang-You Xia, Wen-Long Wang, Shan-Heng Wang, Yan-Lan Huang, Shang Shan

**Affiliations:** a Department of Chemistry, Zunyi Normal College, People’s Republic of China; b College of Chemical Engineering and Materials Science, Zhejiang University of Technology, People’s Republic of China

## Abstract

In the mol­ecular structure of the title compound, C_15_H_15_NO_3_, the two benzene rings are twisted with respect to each other, making a dihedral angle of 75.11 (10)°. In the amide fragment, the C=O and C—N bond distances are 1.248 (3) and 1.321 (3) Å, respectively, indicating electron delocalization. A partially ovelapped arrangement between parallel hydroxy­methoxy­benzene rings is observed in the crystal structure, and the face-to-face distance of 3.531 (16) Å suggests the existence of weak π–π stacking. N—H⋯O and O—H⋯O hydrogen bonding is also present in the crystal structure.

## Related literature

The title compound was obtained during an investigation of capsaicin and its derivatives. For the biological activity of capsaicin, see: Kaga *et al.* (1989 ▶). For related structures, see: Luo & Huang (2004 ▶); Tong *et al.* (2008 ▶).
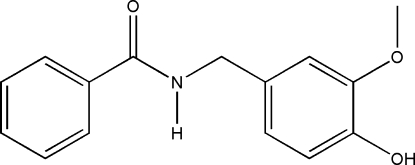


## Experimental

### 

#### Crystal data

C_15_H_15_NO_3_
*M* *_r_* = 257.28
Monoclinic, 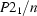

*a* = 7.2292 (18) Å
*b* = 21.057 (5) Å
*c* = 9.031 (2) Å
β = 106.849 (12)°
*V* = 1315.7 (5) Å^3^
*Z* = 4
Mo *K*α radiation
μ = 0.09 mm^−1^
*T* = 294 K
0.40 × 0.28 × 0.26 mm

#### Data collection

Rigaku R-AXIS RAPID IP diffractometer
Absorption correction: none
8839 measured reflections
2353 independent reflections
1304 reflections with *I* > 2σ(*I*)
*R*_int_ = 0.060

#### Refinement

*R*[*F* ^2^ > 2σ(*F* ^2^)] = 0.064
*wR*(*F* ^2^) = 0.186
*S* = 1.00
2353 reflections
182 parameters
H atoms treated by a mixture of independent and constrained refinement
Δρ_max_ = 0.22 e Å^−3^
Δρ_min_ = −0.21 e Å^−3^

### 

Data collection: *PROCESS-AUTO* (Rigaku, 1998 ▶); cell refinement: *PROCESS-AUTO*; data reduction: *CrystalStructure* (Rigaku/MSC, 2002 ▶); program(s) used to solve structure: *SIR92* (Altomare *et al.*, 1993 ▶); program(s) used to refine structure: *SHELXL97* (Sheldrick, 2008 ▶); molecular graphics: *ORTEP-3 for Windows* (Farrugia, 1997 ▶); software used to prepare material for publication: *WinGX* (Farrugia, 1999 ▶).

## Supplementary Material

Crystal structure: contains datablocks I, global. DOI: 10.1107/S1600536809027500/xu2556sup1.cif

Structure factors: contains datablocks I. DOI: 10.1107/S1600536809027500/xu2556Isup2.hkl

Additional supplementary materials:  crystallographic information; 3D view; checkCIF report

## Figures and Tables

**Table 1:** Hydrogen-bond geometry (Å, °)

*D*—H⋯*A*	*D*—H	H⋯*A*	*D*⋯*A*	*D*—H⋯*A*
N1—H1*N*⋯O3^i^	0.85 (3)	2.42 (3)	3.145 (6)	143 (2)
O3—H3*A*⋯O1^ii^	0.98 (4)	1.80 (4)	2.745 (5)	160 (5)

Symmetry codes: (i) 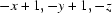; (ii) 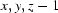.

**Fig. 1.**
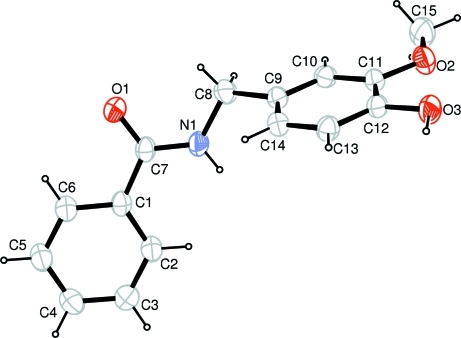
The molecular structure of the title compound with 40% probability displacement (arbitrary spheres for H atoms).

**Fig. 2.**
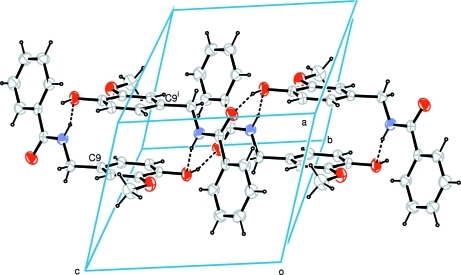
The unit cell packing diagram showing π-π stacking between C9-benzene rings [symmetry code: (i) 1 - x, 1 - y, -z]. Dashed lines indicate the hydrogen bonding.

## supplementary crystallographic information

### Comment

Capsaicin, a pungent principle of capsicums, has been known to exhibit a variety of biological activities, including recent findings concerning its mutagenicity (Kaga *et al.* 1989). During the investigation on syntheses of capsaicin and its derivatives, the title compound has recently been obtained and its crystal structure is reported here.

In the molecular structure of the title compound (Fig. 1), two benzene rings are twisted to each other with a dihedral angle of 75.11 (10)°, which is similar to 72.1 (2)° found in *N*-2-chlorobenzylbenzamide (Luo & Huang, 2004) but is somewhat larger than 56.32 (17)° found in *N*-(4-Cyanobenzyl)benzamide (Tong *et al.* 2008). The amide flagment is nearly coplanar with the C1-benzene ring [dihedral angle 5.0 (4)°]. The C7═O1 and C7—N1 bond distances are 1.248 (3) and 1.321 (3) Å, respectively, showing the electron delocalization in the amide fragment.

A partially ovelapped arrangement between parallel C9-benzene and C9^i^-benzene rings is observed in the crystal structure (Fig. 2), the face-to-face distance of 3.531 (16) Å suggests the existence of weak π-π stacking between them [symmetry code: (i) 1 - *x*,1 - *y*,-*z*]. The N—H···O and O—H···O hydrogen bonding is present in the crystal structure (Table 1 and Fig. 2), which helps to stabilize the crystal structure.

### Experimental

4-Hydroxy-3-methoxy benzylamine HCl salt (4.7 g, 25 mmol) and dimethylformamide (25 ml) were added to a 100 ml 3-necked flask equipped with an additional funnel, a thermometer and a magnetic stirrer. Water solution (10 ml) of NaOH (2.0 g) was added at room temperature. The mixture was stirred at 308 K for 30 min and then cooled to 273 K. An ether solution (10 ml) of benzoyl chloride (3.5 g, 25 mmol) was added dropwise at about 273 K over 20 min. After stirred for 2 h at room temperature the mixture was poured into water, and then extracted with ethyl acetate. The ethyl acetate extract was washed with 1 *M* HCl followed by saturated NaHCO_3_ and brine. The extract was then dried over anhydrous Na_2_SO_4_ and filtered. Solvents were removed under vacuum at about 308 K to give a solid crude. Recrystallization was performed twice with an absolute ethyl acetate to obtain single crystals of the title compound.

### Refinement

Hydroxy and imino H atoms were located in a difference Fourier map and were refined isotropically. Methyl H atoms were placed in calculated positions with C—H = 0.96 Å and torsion angle was refined to fit the electron density, *U*_iso_(H) = 1.5*U*_eq_(C). Other H atoms were placed in calculated positions with C—H = 0.93 (aromatic) and 0.97 Å (methylene), and refined in riding mode with *U*_iso_(H) = 1.2*U*_eq_(C).

### Crystal data

**Table:**

C_15_H_15_NO_3_	*F*(000) = 544
*M**_r_* = 257.28	*D*_x_ = 1.299 Mg m^−^^3^
Monoclinic, *P*2_1_/*n*	Mo *K*α radiation, λ = 0.71073 Å
Hall symbol: -P 2yn	Cell parameters from 4326 reflections
*a* = 7.2292 (18) Å	θ = 2.2–25.1°
*b* = 21.057 (5) Å	µ = 0.09 mm^−^^1^
*c* = 9.031 (2) Å	*T* = 294 K
β = 106.849 (12)°	Prism, colorless
*V* = 1315.7 (5) Å^3^	0.40 × 0.28 × 0.26 mm
*Z* = 4	

### Data collection

**Table:**

Rigaku R-AXIS RAPID IP diffractometer	1304 reflections with *I* > 2σ(*I*)
Radiation source: fine-focus sealed tube	*R*_int_ = 0.060
graphite	θ_max_ = 25.2°, θ_min_ = 1.9°
Detector resolution: 10.0 pixels mm^-1^	*h* = −8→8
ω scans	*k* = −25→25
8839 measured reflections	*l* = −10→10
2353 independent reflections	

### Refinement

**Table:**

Refinement on *F*^2^	Secondary atom site location: difference Fourier map
Least-squares matrix: full	Hydrogen site location: inferred from neighbouring sites
*R*[*F*^2^ > 2σ(*F*^2^)] = 0.064	H atoms treated by a mixture of independent and constrained refinement
*wR*(*F*^2^) = 0.186	*w* = 1/[σ^2^(*F*_o_^2^) + (0.0865*P*)^2^] where *P* = (*F*_o_^2^ + 2*F*_c_^2^)/3
*S* = 1.00	(Δ/σ)_max_ < 0.001
2353 reflections	Δρ_max_ = 0.22 e Å^−^^3^
182 parameters	Δρ_min_ = −0.21 e Å^−^^3^
0 restraints	Extinction correction: *SHELXL97* (Sheldrick, 2008), Fc^*^=kFc[1+0.001xFc^2^λ^3^/sin(2θ)]^-1/4^
Primary atom site location: structure-invariant direct methods	Extinction coefficient: 0.024 (4)

### Special details

**Table:**

Geometry. All e.s.d.'s (except the e.s.d. in the dihedral angle between two l.s. planes) are estimated using the full covariance matrix. The cell e.s.d.'s are taken into account individually in the estimation of e.s.d.'s in distances, angles and torsion angles; correlations between e.s.d.'s in cell parameters are only used when they are defined by crystal symmetry. An approximate (isotropic) treatment of cell e.s.d.'s is used for estimating e.s.d.'s involving l.s. planes.
Refinement. Refinement of *F*^2^ against ALL reflections. The weighted *R*-factor *wR* and goodness of fit *S* are based on *F*^2^, conventional *R*-factors *R* are based on *F*, with *F* set to zero for negative *F*^2^. The threshold expression of *F*^2^ > σ(*F*^2^) is used only for calculating *R*-factors(gt) *etc*. and is not relevant to the choice of reflections for refinement. *R*-factors based on *F*^2^ are statistically about twice as large as those based on *F*, and *R*- factors based on ALL data will be even larger.

### Fractional atomic coordinates and isotropic or equivalent isotropic displacement parameters (Å^2^)

**Table:**

	*x*	*y*	*z*	*U*_iso_*/*U*_eq_	
N1	0.4418 (4)	0.57597 (12)	0.3750 (3)	0.0609 (8)	
O1	0.2826 (3)	0.61857 (10)	0.5293 (2)	0.0704 (7)	
O2	0.2344 (3)	0.40542 (9)	−0.1741 (2)	0.0699 (7)	
O3	0.2085 (3)	0.50638 (11)	−0.3439 (2)	0.0681 (7)	
C1	0.5991 (4)	0.65751 (12)	0.5602 (3)	0.0501 (7)	
C2	0.7756 (4)	0.65520 (14)	0.5270 (3)	0.0624 (8)	
H2	0.7941	0.6257	0.4560	0.075*	
C3	0.9237 (5)	0.69650 (15)	0.5988 (4)	0.0730 (10)	
H3	1.0410	0.6947	0.5760	0.088*	
C4	0.8970 (5)	0.74018 (16)	0.7039 (4)	0.0774 (10)	
H4	0.9962	0.7680	0.7517	0.093*	
C5	0.7239 (5)	0.74283 (16)	0.7384 (4)	0.0789 (10)	
H5	0.7063	0.7722	0.8101	0.095*	
C6	0.5766 (5)	0.70172 (13)	0.6662 (4)	0.0656 (9)	
H6	0.4597	0.7039	0.6895	0.079*	
C7	0.4317 (4)	0.61524 (13)	0.4864 (3)	0.0526 (8)	
C8	0.2814 (5)	0.53450 (15)	0.2939 (3)	0.0640 (9)	
H8A	0.1614	0.5516	0.3050	0.077*	
H8B	0.3004	0.4927	0.3413	0.077*	
C9	0.2655 (4)	0.52835 (13)	0.1243 (3)	0.0512 (8)	
C10	0.2606 (4)	0.46920 (13)	0.0575 (3)	0.0530 (8)	
H10	0.2709	0.4331	0.1186	0.064*	
C11	0.2408 (4)	0.46267 (13)	−0.0981 (3)	0.0508 (8)	
C12	0.2250 (4)	0.51616 (14)	−0.1907 (3)	0.0511 (7)	
C13	0.2304 (4)	0.57554 (13)	−0.1242 (3)	0.0569 (8)	
H13	0.2204	0.6117	−0.1851	0.068*	
C14	0.2505 (4)	0.58145 (14)	0.0317 (3)	0.0576 (8)	
H14	0.2540	0.6217	0.0750	0.069*	
C15	0.2403 (6)	0.34901 (15)	−0.0863 (4)	0.0872 (11)	
H15A	0.3633	0.3459	−0.0092	0.131*	
H15B	0.2221	0.3128	−0.1537	0.131*	
H15C	0.1393	0.3502	−0.0369	0.131*	
H1N	0.546 (4)	0.5707 (13)	0.350 (3)	0.063 (10)*	
H3A	0.204 (7)	0.548 (2)	−0.393 (5)	0.148 (18)*	

### Atomic displacement parameters (Å^2^)

**Table:**

	*U*^11^	*U*^22^	*U*^33^	*U*^12^	*U*^13^	*U*^23^
N1	0.0560 (17)	0.0776 (18)	0.0497 (16)	−0.0013 (14)	0.0162 (14)	−0.0173 (13)
O1	0.0796 (16)	0.0755 (15)	0.0682 (15)	−0.0099 (12)	0.0404 (12)	−0.0126 (11)
O2	0.0938 (16)	0.0555 (13)	0.0618 (14)	−0.0044 (11)	0.0247 (12)	−0.0115 (11)
O3	0.0854 (16)	0.0777 (16)	0.0449 (13)	−0.0053 (12)	0.0247 (11)	−0.0056 (11)
C1	0.0671 (19)	0.0473 (16)	0.0358 (15)	0.0076 (14)	0.0149 (14)	0.0045 (13)
C2	0.068 (2)	0.0613 (19)	0.0585 (19)	0.0032 (16)	0.0192 (16)	−0.0100 (15)
C3	0.069 (2)	0.079 (2)	0.074 (2)	−0.0035 (18)	0.0259 (18)	−0.0077 (18)
C4	0.082 (3)	0.076 (2)	0.072 (2)	−0.0169 (19)	0.0189 (19)	−0.0176 (19)
C5	0.099 (3)	0.068 (2)	0.075 (2)	−0.010 (2)	0.034 (2)	−0.0211 (18)
C6	0.073 (2)	0.0619 (19)	0.065 (2)	−0.0006 (16)	0.0253 (17)	−0.0104 (16)
C7	0.068 (2)	0.0524 (17)	0.0390 (16)	0.0050 (15)	0.0181 (15)	0.0010 (13)
C8	0.069 (2)	0.076 (2)	0.0461 (18)	−0.0121 (16)	0.0144 (15)	−0.0081 (16)
C9	0.0497 (17)	0.0587 (18)	0.0450 (17)	−0.0037 (13)	0.0134 (14)	−0.0059 (14)
C10	0.0529 (18)	0.0561 (18)	0.0498 (18)	−0.0038 (13)	0.0144 (14)	0.0005 (14)
C11	0.0516 (18)	0.0556 (17)	0.0453 (17)	−0.0030 (13)	0.0141 (13)	−0.0088 (14)
C12	0.0521 (18)	0.0618 (18)	0.0391 (17)	−0.0039 (14)	0.0129 (13)	−0.0042 (14)
C13	0.065 (2)	0.0556 (18)	0.0521 (19)	0.0024 (14)	0.0206 (15)	0.0029 (15)
C14	0.0630 (19)	0.0570 (18)	0.0545 (19)	−0.0045 (14)	0.0198 (15)	−0.0098 (15)
C15	0.118 (3)	0.056 (2)	0.099 (3)	−0.004 (2)	0.050 (2)	−0.0084 (19)

### Geometric parameters (Å, °)

**Table:**

N1—C7	1.321 (3)	C5—H5	0.9300
N1—C8	1.467 (4)	C6—H6	0.9300
N1—H1N	0.85 (3)	C8—C9	1.508 (4)
O1—C7	1.248 (3)	C8—H8A	0.9700
O2—C11	1.382 (3)	C8—H8B	0.9700
O2—C15	1.422 (4)	C9—C10	1.380 (4)
O3—C12	1.370 (3)	C9—C14	1.382 (4)
O3—H3A	0.98 (4)	C10—C11	1.377 (4)
C1—C6	1.378 (4)	C10—H10	0.9300
C1—C2	1.393 (4)	C11—C12	1.388 (4)
C1—C7	1.494 (4)	C12—C13	1.382 (4)
C2—C3	1.386 (4)	C13—C14	1.379 (4)
C2—H2	0.9300	C13—H13	0.9300
C3—C4	1.374 (4)	C14—H14	0.9300
C3—H3	0.9300	C15—H15A	0.9600
C4—C5	1.375 (4)	C15—H15B	0.9600
C4—H4	0.9300	C15—H15C	0.9600
C5—C6	1.381 (4)		
			
C7—N1—C8	122.9 (3)	C9—C8—H8A	109.2
C7—N1—H1N	122 (2)	N1—C8—H8B	109.2
C8—N1—H1N	114.9 (19)	C9—C8—H8B	109.2
C11—O2—C15	117.4 (2)	H8A—C8—H8B	107.9
C12—O3—H3A	108 (3)	C10—C9—C14	118.6 (3)
C6—C1—C2	118.2 (3)	C10—C9—C8	120.4 (3)
C6—C1—C7	117.9 (3)	C14—C9—C8	121.0 (3)
C2—C1—C7	123.9 (2)	C11—C10—C9	121.2 (3)
C3—C2—C1	120.6 (3)	C11—C10—H10	119.4
C3—C2—H2	119.7	C9—C10—H10	119.4
C1—C2—H2	119.7	C10—C11—O2	124.9 (3)
C4—C3—C2	119.9 (3)	C10—C11—C12	120.0 (3)
C4—C3—H3	120.0	O2—C11—C12	115.1 (2)
C2—C3—H3	120.0	O3—C12—C13	123.9 (3)
C3—C4—C5	120.2 (3)	O3—C12—C11	117.0 (3)
C3—C4—H4	119.9	C13—C12—C11	119.1 (3)
C5—C4—H4	119.9	C14—C13—C12	120.4 (3)
C4—C5—C6	119.7 (3)	C14—C13—H13	119.8
C4—C5—H5	120.2	C12—C13—H13	119.8
C6—C5—H5	120.2	C13—C14—C9	120.8 (3)
C1—C6—C5	121.4 (3)	C13—C14—H14	119.6
C1—C6—H6	119.3	C9—C14—H14	119.6
C5—C6—H6	119.3	O2—C15—H15A	109.5
O1—C7—N1	121.0 (3)	O2—C15—H15B	109.5
O1—C7—C1	119.3 (2)	H15A—C15—H15B	109.5
N1—C7—C1	119.7 (3)	O2—C15—H15C	109.5
N1—C8—C9	112.0 (2)	H15A—C15—H15C	109.5
N1—C8—H8A	109.2	H15B—C15—H15C	109.5
			
C6—C1—C2—C3	−0.1 (4)	N1—C8—C9—C14	−54.6 (4)
C7—C1—C2—C3	179.2 (3)	C14—C9—C10—C11	−0.1 (4)
C1—C2—C3—C4	0.0 (5)	C8—C9—C10—C11	178.2 (2)
C2—C3—C4—C5	0.3 (5)	C9—C10—C11—O2	−179.9 (3)
C3—C4—C5—C6	−0.5 (5)	C9—C10—C11—C12	−0.1 (4)
C2—C1—C6—C5	−0.1 (4)	C15—O2—C11—C10	2.9 (4)
C7—C1—C6—C5	−179.5 (3)	C15—O2—C11—C12	−176.9 (3)
C4—C5—C6—C1	0.4 (5)	C10—C11—C12—O3	179.1 (2)
C8—N1—C7—O1	0.5 (4)	O2—C11—C12—O3	−1.1 (4)
C8—N1—C7—C1	−178.1 (2)	C10—C11—C12—C13	0.3 (4)
C6—C1—C7—O1	−4.3 (4)	O2—C11—C12—C13	−179.9 (2)
C2—C1—C7—O1	176.4 (3)	O3—C12—C13—C14	−178.9 (3)
C6—C1—C7—N1	174.4 (2)	C11—C12—C13—C14	−0.2 (4)
C2—C1—C7—N1	−5.0 (4)	C12—C13—C14—C9	0.0 (5)
C7—N1—C8—C9	142.7 (3)	C10—C9—C14—C13	0.2 (4)
N1—C8—C9—C10	127.1 (3)	C8—C9—C14—C13	−178.1 (3)

### Hydrogen-bond geometry (Å, °)

**Table:**

*D*—H···*A*	*D*—H	H···*A*	*D*···*A*	*D*—H···*A*
N1—H1N···O3^i^	0.85 (3)	2.42 (3)	3.145 (6)	143 (2)
O3—H3A···O1^ii^	0.98 (4)	1.80 (4)	2.745 (5)	160 (5)

Symmetry codes: (i) −*x*+1, −*y*+1, −*z*; (ii) *x*, *y*, *z*−1.
